# Vitamin D intake, blood vitamin D levels, and the risk of breast cancer: a dose-response meta-analysis of observational studies

**DOI:** 10.18632/aging.102597

**Published:** 2019-12-28

**Authors:** Dingli Song, Yujiao Deng, Kang Liu, Linghui Zhou, Na Li, Yi Zheng, Qian Hao, Si Yang, Ying Wu, Zhen Zhai, Hongtao Li, Zhijun Dai

**Affiliations:** 1 Department of Breast Surgery, The First Affiliated Hospital, College of Medicine, Zhejiang University, Hangzhou, China; 2 Department of Oncology, The Second Affiliated Hospital of Xi’an Jiaotong University, Xi’an, China; 3 Department of Hepatobiliary Surgery, The First Affiliated Hospital of Xi'an Jiaotong University, Xi'an, China; 4 Department of Breast Head and Neck Surgery, The 3rd Affiliated Teaching Hospital of Xinjiang Medical University (Affiliated Tumor Hospital), Urumqi, China

**Keywords:** vitamin D, dose-response, breast cancer risk, menopause, meta-analysis

## Abstract

Epidemiological studies have indicated that blood vitamin D levels are linked to cancer. Here we conducted a dose–response meta-analysis based on published observational studies to evaluate the association of vitamin D intake and blood vitamin D levels with breast cancer susceptibility. PubMed, EMBASE, and Web of Science databases were searched up to January 2019. The pooled odds ratio (OR) and 95% confidence intervals (CIs) were extracted to estimate the risk. We identified 70 relevant studies on blood vitamin D levels (50 studies) and vitamin D intake (20 studies), respectively. Linear and nonlinear trend analyses were performed and showed that an increase in blood vitamin D levels by 5 nmol/l was associated with a 6% decrease in breast cancer risk (OR = 0.94, 95% CI = 0.93–0.96). Similar results were obtained for premenopausal (OR = 0.96, 95% CI = 0.93–0.99) and postmenopausal women (OR = 0.96, 95% CI = 0.94–0.98). The pooled OR of breast cancer risk for a 400IU/day increase in vitamin D intake was 0.97 (95% CI = 0.92–1.02). In conclusion, we found that breast cancer risk was inversely related to blood vitamin D levels; however, no significant association was observed in vitamin D intake.

## INTRODUCTION

Breast cancer is the most common form of malignancy and the main cause of cancer-related death among women worldwide [1]. Epidemiological studies revealed that the incidence of breast cancer has been increasing globally since the end of the 1970s [2]. According to a recent study conducted by the American Cancer Society, the most three commonly diagnosed cancer in 2019 are breast, lung, and colorectal cancer. Furthermore, breast cancer accounts for 30% of the all newly diagnosed cancer cases in women [3].

Vitamin D is a steroid derivative, and plays a key role in promoting bone growth. Epidemiological studies have shown that low serum vitamin D levels was linked to a higher risk of colon and bladder cancer, and higher circulating concentration of 25(OH)D decreased the risk of renal cell carcinoma [4–6]. Additionally, an anti-cancer effect on vitamin D against breast, prostate, and colorectal cancers have been reported [7]. The review has clarified that calcitriol, the product of vitamin D, was involved in the proliferation, apoptosis, differentiation, inflammation, invasion, angiogenesis and metastasis of tumor by regulating various signaling pathways, which may affect the development and growth of tumor [8]. Previous experimental studies has revealed that the active metabolite of vitamin D, 1, 25(OH)_2_D, inhibited breast cancer progression and metastasis by inducing apoptosis, reducing cell growth and angiogenesis [9]. Since the 1970s, numerous observational studies have discussed the relationship between vitamin D and the risk of breast cancer [10]; however, the results of individual studies do not show a similar association. Although recently published meta-analysis and reviews have focused on the relationship between blood vitamin D levels and vitamin D intake with breast cancer risk [11–15], the findings remain controversial. Therefore, based on prospective cohort and case-control studies, we conducted a dose-response meta-analysis to systematically assess the relationship of vitamin D intake and vitamin D levels with the risk of breast cancer.

## RESULTS

### Literature selection and study characteristics

**Figure 1:**
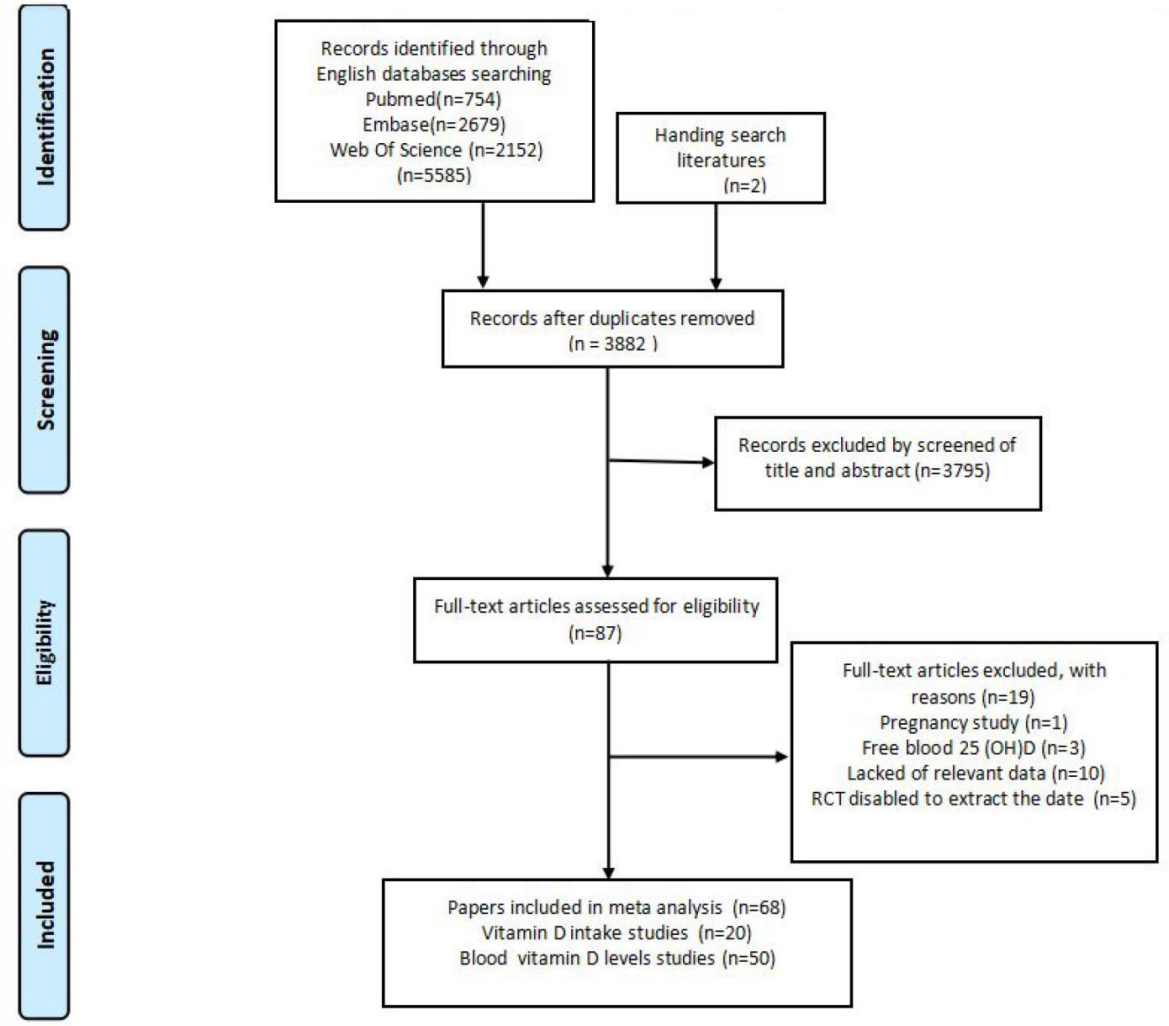
**Flowchart of included studies for the meta-analysis.**

The flow chart of the selection of publications from the existing literature is shown in Figure 1. Firstly, 5587 articles were searched through the databases as well as through hand searching. Next, 1705 articles were excluded for duplication; 3795 articles were excluded after reading the titles and abstracts for the lack of relevance; 10 articles did not contain the relevant data; three articles measured blood vitamin D levels in pg/ml or pmol/l; five articles disabled the extraction data; and one article was associated with pregnancy. Additional there are two studies, O’ Brien et al [16] and Fedirko et al [17], provide data on blood vitamin D and vitamin D intake. So we went through the full texts of 68 articles. Finally, a total of 68 articles that contained 70 observational studies (case-control or cohort) were eligible for the analysis. The characteristics of the 68 selected publications are summarized in Table 1 and Supplementary Table 1.

**Table 1:** Characteristics of prospective studies included in the meta-analysis of vitamin D intake and breast cancer risk.

**Author**	**Country**	**Study type**	**Follow-up period (year)**	**Age (year)**	**No. of cases/controls/ persons**	**Vitamin D Intake (IU/day)^a^**	**Adjusted OR(95%CI)^b^**	**Adjustment factors**
O’Brien et al, 2017	USA	Cohort	5	35-74	1699/49044	Total ≥600 vs <200	0.90 (0.78–1.05)	Age, BMI, race, education, menopausal status, current birth control use, physical activity, hormone therapy type, current alcohol use, osteoporosis, total energy intake, parity, and a BMI× menopausal status interaction term
Abbas et al, 2013	Europe	Cohort	8.8	50.2	7760/319985	Dietary ≥218.4 vs <74	1.04 (0.94–1.14)	No-fat, no-alcohol energy, fat, alcohol consumption, weight, height, smoking status, menopausal status, physical activity, age at menarche, education level and current use of contraceptives or hormones
Rollison et al, 2012	USA	Case- control	1999-2004	24-79	2318/2521	Dietary 7.0-122.5 vs 308.6-1362.7	1.28 (1.09-1.5)	Age
Fedirko et al, 2012	Mexico	Case- control	2004-2007	35-69	570/638	Dietary >111.8 vs ≤65	0.69 (0.47–1.00)	SES, BMI, alcohol consumption, height, parity/number of children born alive, age at first full term pregnancy, family history of breast cancer, breast feeding, use of hormone for menopause, physical activity index, total energy intake, and menopausal status
Edvardsen et al, 2011	Norway	Cohort	1997-2007	40-70	844/41758	Total ≥832 vs ≤108	1.07 (0.87–1.32)	Age at entry, BMI, height, menopausal status, HRT use, use of oral contraceptives, mothers’ history of breast cancer, frequency of mammography, combined parity and age at first birth and daily intake of alcohol.
Kawase et al, 2010	Japan	Case- control	2001-2005	20-79	1803/3606	Dietary 266-1400 vs 80-114	0.76 (0.63–0.9)	Age, BMI, menopausal status, smoking habit, drinking habit, physical activity, family history of breast cancer in a first degree relative, age at menarche, parity, hormone use, total nonalcohol energy, and referral pattern
Anderson et al, 2010	Canada	Case- control	2002-2003	25-74	3101/3471	Total ≥600 vs <100	0.99 (0.78-1.26)	Age, BMI, education, age at menarche, age at first live birth, parity, menopausal status, smoking, relative energy intake, breast cancer in first degree, moderate physical activity, time spent outdoors, total calcium intake, and total vitamin D intake
Lee et al, 2010	China	Case- control	2004-2005	Cases 52.5 Controls 48.9	200/200	Total 428-1148 vs 6.8-125.6	0.52 (0.25–1.07)	Age, BMI, education, parity, use of HRT, total energy intake, sunlight exposure, menopausal status, and homocysteine.
Engel et al, 2010	French	Cohort	10.4	41.8-72	2871/67721	Dietary >113 vs <80	0.94 (0.86–1.03)	BMI, age at menopause, age at menarche, physical activity, parity, use of menopausal, use of HRT, alcohol intake, daily calcium intake, calcium supplement, energy intake without alcohol, university degree, previous family history of breast cancer, previous personal history of benign breast disease, previous history of mammographic exam, sun burn resistance, menopausal status, and skin complexion.
Kuper et al, 2009	Sweden	cohort	1991-2003 (12.9)	30-49	840/41889	Dietary Q4 vs Q1	0.90 (0.80–1.10)	BMI, parity, age at first birth, age at menarche, use of hormonal contraceptives, consumption of alcohol, breast-feeding, education, family history of breast cancer, physical activity, and smoking.
Rossi et al, 2008	Italy	Case- control	1991-1994	23-74	2569/2588	Total >190.4 vs <60.4	0.76 (0.58–1.00)	age, parity, age at menarche, study center, education, total energy intake, menopausal status, vegetable and fruit consumption, calcium, b-carotene, vitamin E, flavones, and flavonol intake
Abbas et al, 2007	German	Case- control	1992-1995	24-50	278/666	Dietary ≥200 vs < 80	0.50 (0.26–0.96)	BMI, age at menarche, energy intake, duration of breast feeding, first-degree family history, number of births, nonalcohol, alcohol consumption, and mineral and vitamin supplements
Robien et al, 2007	USA	Cohort	1986-2004	50-70	2440/34321	Total ≥800 vs <400	0.90 (0.78–1.04)	Age, BMI, smoking status, age at menarche, age at menopause, first degree relative with breast cancer, estrogen use, age at first live birth, number of live births, education category, activity level, live on a farm, mammogram history, and daily energy, fat, and alcohol intake.
Lin et al, 2007	USA	Cohort	10	55.2	Cases Pre276/Post743 Persons Pre10578/Post20909	Total ≥548 vs <162	Pre 0.65 (0.42-1.00) Post 1.30 (0.97-1.73)	Age, BMI, randomized treatment assignment, physical activity, family history of breast cancer in a first-degree relative, history of benign breast disease, age at menarche, parity, age at first birth, multivitamin use, smoking status, alcohol consumption, total energy intake, age at menopause, and baseline postmenopausal hormone therapy.
McCullough et al,2005	USA	Cohort	1992-2001	50-74	2855/68567	Total ≥700 vs ≤100	0.94 (0.80-1.10)	Age, energy, history of breast cyst, family history of breast cancer, height, weight gain since age 18, alcohol use, race, age at menopause, age at first birth and number of live births, education, mammography history, and hormone therapy.
Frazier et al, 2004	USA	Cohort	1989-1998	34-51	361/47517	Total 591 vs 159.6	0.92 (0.66-1.27)	Age, BMI, time period, height, parity and age at first birth, age at menarche, family history of breast cancer, history of benign breast disease, menopausal status, alcohol intake, energy, oral contraceptive use, and weight gain since age 18.
Shin et al, 2002	USA	Cohort	1980-1996	46.7	Pre827/Post2345/ 88691	Total >500 vs ≤150	Pre 0.89 (0.68-1.15) Post 0.93 (0.80-1.08)	Age, BMI, time period, physical activity, history of benign breast disease, family history of breast cancer, height, weight change, age at menarche, parity, age at first birth, alcohol intake, total energy intake, total fat intake, glycemic index, β-carotene intake, and total active vitamin E intake.
Levi et al, 2000	Switzerland	Case- control	1993-1999	23-74	289/442	Total 108000 vs 56000	1.43 (0.90–2.26)	Age, BMI, education, parity, menopausal status, total energy intake, and alcohol drinking
John et al, 1999	USA	Cohort	1971-1992 (17.3)	25-74	179/4747	Dietary ≥200 vs <100	0.85 (0.59–1.24)	Age, BMI, education, age at menarche, age at menopause, frequency of alcohol consumption, physical activity, and calcium intake
Potischman et al,1999	USA	Case- control	1990-1992	20-44 years	568/1451	Total ≥400 vs 0	0.98 (0.8–1.2)	Age at diagnosis, study site, ethnicity, combination age at first birth and parity, of oral contraceptive use, smoking, education and alcohol consumption.

Abbreviations: OR=odds ratio; CI=confidence interval; BMI=body mass index (kg/m^2); HRT=hormone replacement therapy; HT=hormone therapy; POST=postmenopausal; PRE=premenopausal.

a. Vitamin D intake levels in ug/day were converted to IU/day using the conversion factor, 1ug/d=40IU/day.

b. The ORs of all studies used the lowest category of vitamin D intake levels as a reference in the meta-analysis.

For the association of vitamin D intake with breast cancer risk, there are 20 relevant studies, including 11 prospective cohort studies [16, 18–27] consisting 24040 cases, and 9 case-control studies [17, 28–35] consisting 11696 cases and 15583 controls. Among these, nine studies were performed in the US [16, 18–21, 25, 26, 28, 33], seven in Europe [22–24, 27, 29, 34, 35], two in Asia [31, 32], one in Canada [30] and one in Mexico [17]. There were seven studies [17, 18, 21, 22, 24, 31, 32] that provided risk estimates which were stratified by menopausal status in the 20 studies. The risk estimates of most of the studies were adjusted for potential confounders, including age, body mass index (BMI), education level, and physical activity. The adjusted confounding factors are shown in Table 1.

We identified 50 prospective studies on the association of blood vitamin D levels and breast cancer risk; the six cohort studies [36–41] consisting of 2257 incident cases, and 44 case-control studies [16, 17, 42–83] consisting 29095 cases and 53060 controls were included. Among these, 16 studies were conducted in the Europe [36, 38–40, 43, 44, 47, 50–57], fifteen in the US [16, 19, 37, 41, 42, 45, 46, 48, 58–63, 82], twelve in Asia [64–75], two in Canada [76, 77], one each in Austria [78], Mexico [17], and Brazil [79]. In addition, two studies [80, 81] contained mixed population in Europe and in the US. The menopausal status was categorized as premenopausal, postmenopausal, or mixed. Overall, nine studies [16, 17, 48, 50–53, 62, 64] assessed risk estimates based on participants’ menopausal status and twelve studies [43, 50, 57, 59, 65, 68, 71, 72, 77–79, 83] provided unadjusted results. The majority of the studies were adjusted for potential confounders including age, BMI, race, education level and time at blood collection.

### Overall analyses

### Vitamin D intake and breast cancer risk

**Figure 2:**
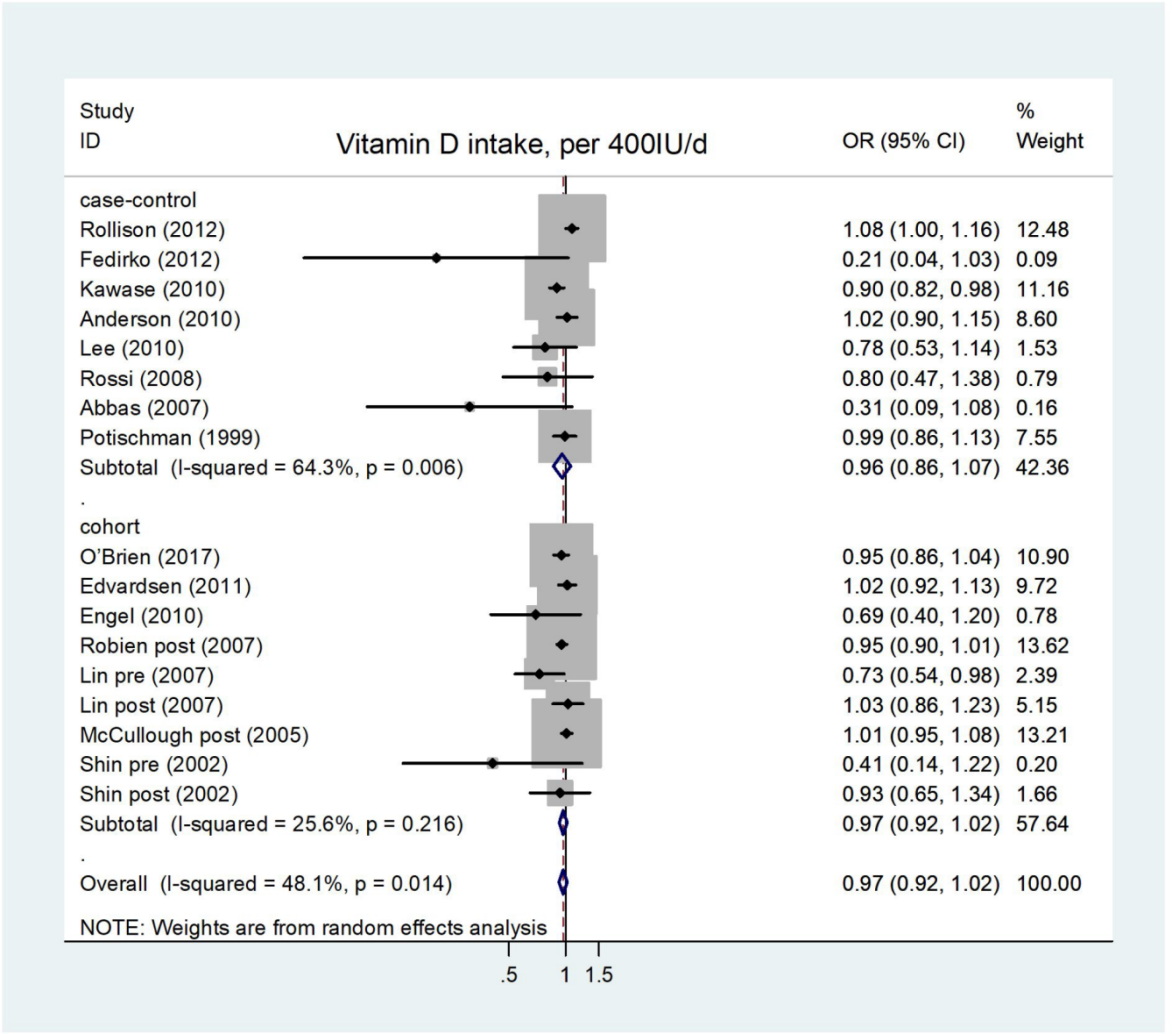
**Forest plot of meta-analysis of the association between vitamin D intake increment (per 400IU/d) and breast cancer risk.** Abbreviations: OR, odds ratio; CI, confidence interval.

The pooled OR of breast cancer risk for the highest versus lowest category of vitamin D intake was 0.94 (95% CI = 0.88–1.00), with an evidence of heterogeneity I^2^ = 57.2%, P = 0.000 (Supplementary Figure 1). No publication bias was found after visual inspection of the funnel the plot (Supplementary Figure 3). The summary of estimations for case-control studies were OR = 0.89, 95% CI = 0.73–1.08, I^2^=77.0%, P = 0.228, and for cohort studies were OR=0.95, 95% CI = 0.90–1.00, I^2^ = 16.1%, P = 0.055. Eight case-control studies and seven cohort studies were eligible for the dose-response analysis of the association of vitamin D intake and breast cancer risk. As shown in Figure 2, the random-effects model was used and showed that a 400 IU/day increment in vitamin D intake had no significant effect on occurrence of breast cancer, and the pooled OR were 0.97 (95% CI = 0.92–1.02, I^2^ = 25.6%, P = 0.222) and 0.96 (95% CI = 0.86–1.07, I^2^ = 64.3%, P = 0.427) for cohort studies and case-control studies, respectively. Heterogeneity among studies was statistically significant (I^2^ = 48.1%, P = 0.014). There existed significant publication bias according to Begg's test (P = 0.009) and Egger's test (P = 0.007) (Supplementary Figure 5). In sensitivity analyses, a single study had no influence on the results, and the stable OR in the overall analysis ranged from 0.93–0.98 (Supplementary Figure 7). We failed to identify a significant dose-response relationship between vitamin D intake and breast cancer risk. When the subgroup analysis was stratified by menopausal status, study type, geographical location, and follow-up years, the results were stable; except for Asian studies (OR = 0.97, 95% CI = 0.95–0.99, Table 2) with no significant heterogeneity (I^2^ = 0.0%, P = 0.472), and studies related to premenopuase (OR = 0.79, 95% CI = 0.64–0.96, Table 2) with a significant heterogeneity (I^2^ = 56.1%, P = 0.026). This result suggests that higher vitamin D intake could reduce the risk of breast cancer in Asian women and premenopausal women.

**Table 2:** Subgroup analyses of vitamin D intake and breast cancer.

**Analysis specification**	**No. of studies**	**OR(95% CI)**	***P***	**Heterogeneity**	
				**I^2^**	***p***
Highest vs lowest					
All studies	20	0.94(0.88-1.00)	0.063	57.2%	0
Case-control	9	0.89(0.73-1.08)	0.228	77.0%	0
Cohort	11	0.95(0.90-1.00)	0.055	16.1%	0.281
Increment of 400 IU/d					
All studies	15	0.97(0.92-1.02)	0.201	48.1%	0.014
Case-control	8	0.96(0.86-1.07)	0.427	64.3%	0.006
Cohort	7	0.97(0.92-1.02)	0.222	25.6%	0.216
Menopausal status					
Premenopause	8	0.79(0.64-0.96)	0.021	56.1%	0.026
Postmenopausal	8	0.98(0.94-1.02)	0.243	0	0.631
Geographic location					
Europe	4	0.83(0.60-1.15)	0.257	48.3%	0.122
America	7	0.99(0.93-1.04)	0.599	47.3%	0.056
Asia	2	0.89(0.82-0.97)	0.008	0	0.472
Follow-up duration					
<10 years	9	0.96(0.88-1.05)	0.358	60.9%	0.009
≥10 years	5	0.95(0.88-1.03)	0.245	32%	0.183
Source vitamin D					
Dietary	5	0.90(0.73-1.10)	0.308	78.9%	0.001
Dietary+Supplement	10	0.98(0.94-1.01)	0.185	5.3%	0.393

### Blood vitamin D levels and breast cancer risk

**Figure 3:**
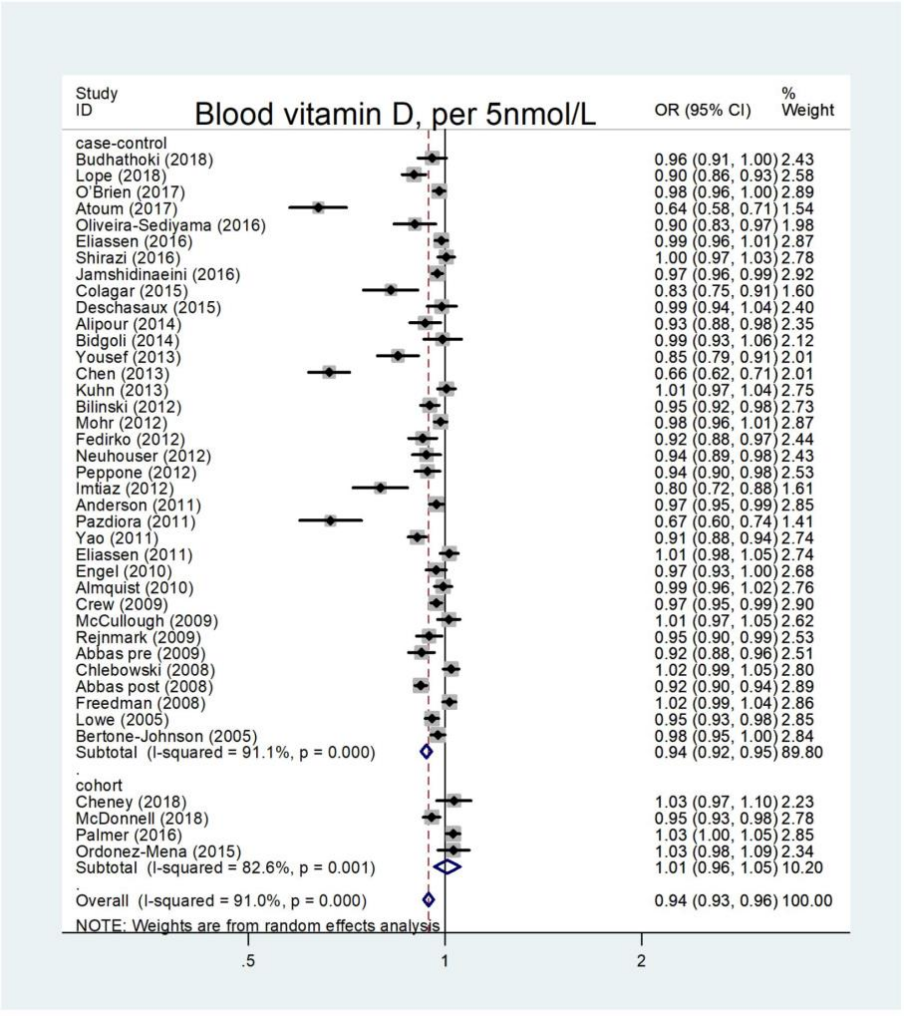
**Forest plot of meta-analysis of the association between blood vitamin D increment (per 5nmol/L) and breast cancer risk.** Abbreviations: OR, odds ratio; CI, confidence interval.

**Figure 4:**
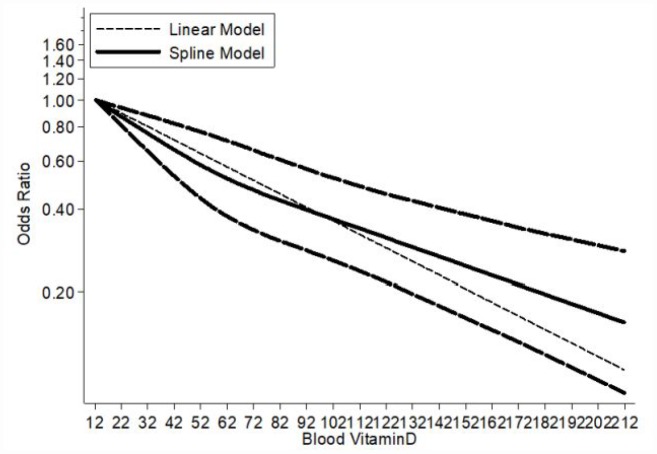
**Dose–response meta-analysis of blood vitamin D and breast cancer risk (linear and nonlinear models).**

**Figure 5:**
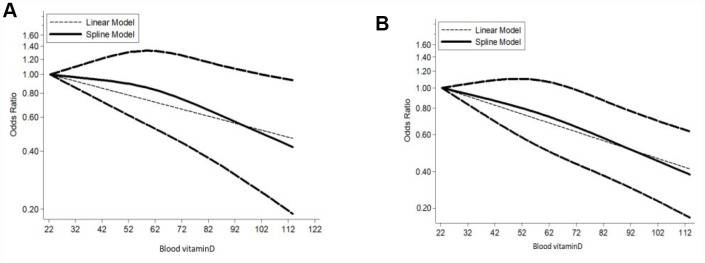
**Dose-response meta-analysis of blood vitamin D and breast cancer risk stratified by menopausal status (linear and nonlinear models).** Note: (**A**) Premenopause; (**B**) Postmenopause.

The summary OR of breast cancer for the highest versus lowest category of blood vitamin D levels was 0.61 (95% CI = 0.53–0.70), with an evidence of heterogeneity I^2^ = 89.3%, P = 0.000 (Supplementary Figure 2). A significant publication bias was observed through the funnel plot (Supplementary Figure 4). The pooled OR for case-control studies was 0.57 (95% CI = 0.48–0.66; I^2^ = 89.9%, P = 0.000), and for cohort studies was 1.17 (95% CI = 0.92–1.48; I^2^ = 31.6%, P = 0.192). Overall, 36 case-control studies and four cohort studies were eligible for the dose-response analysis of the association on blood vitamin D levels with breast cancer risk. The pooled OR for breast cancer risk for a 5 nmol/l increase in blood vitamin D levels was 0.94 (95% CI = 0.93–0.96), with a significant heterogeneity among all studies (I^2^ = 91.0%, P = 0.000) (Figure 3). The summary OR for case-control studies was 0.94 (95% CI = 0.92–0.95) and for cohort studies was 1.01 (95% CI = 0.96–1.05). Begg’s test (P = 0.004) and Egger’s test (P = 0.004) showed a significant publication bias and the funnel plot was asymmetrical (Supplementary Figure 6). The sensitivity analyses indicated that the ORs ranged from 0.96–0.97, and our results were statistically stable (Supplementary Figure 8). 36 eligible case-control studies showed an evidence of a linear association between blood vitamin D levels and breast cancer risk (P_nonlinearity_ = 0.1893) (Figure 4). Subgroup analysis based on menopausal status (Figure 5), showed linear relationship between blood vitamin D levels and breast cancer risk for premenopausal and postmenopausal women (P_nonlinearity_ = 0.2140 and P_nonlinearity_ =0.4900, respectively). The results of subgroup are presented in Table 3. A 5 nmol/l increase in blood vitamin D corresponded to a 16% decrease in breast cancer risk in Asian women.

**Table 3:** Subgroup analyses of blood vitamin D and breast cancer.

**Analysis specification**	**No. of studies**	**OR(95% CI)**	**P**	**Heterogeneity**	
				**I^2**	**p**
Highest vs lowest					
All studies	50	0.61(0.53-0.70)	0	89.3%	0
Case-control	44	0.57(0.48-0.66)	0	89.9%	0
Cohort	6	1.17(0.92-1.48)	0.192	31.6%	0.198
Increment of 5 nmol/l					
All studies	40	0.94(0.93-0.96)	0	91.0%	0
Case-control	36	0.94(0.92-0.95)	0	91.1%	0
Cohort	4	1.01(0.96-1.05)	0.734	82.6%	0.001
Menopausal status					
Premenopause	11	0.96(0.93-0.99)	0.011	68.2%	0
Postmenopausal	15	0.96(0.94-0.98)	0.001	86.4%	0
Geographic location					
Europe	13	0.95(0.92-0.98)	0	89.1%	0
America	14	0.98(0.96-0.99)	0.034	82.4%	0
Asia	9	0.84(0.77-0.92)	0	95.7%	0
Follow-up duration					
<10 years	24	0.94(0.91-0.96)	0	92.4%	0
≥10 years	10	0.99(0.98-1.00)	0.051	26.7%	0.198
Serum or Plasm					
Serum	31	0.93(0.91-0.97)	0	92.80%	0
Plasm	9	0.97(0.96-0.98)	0	50.50%	0.04

## DISCUSSION

Vitamin D is known to be associated with the risk of human cancers, [7, 8, 84] and vitamin D deficiency has been reported to be correlated with colorectal cancer and prostate cancer [85–88]. The recent case-control study suggested that higher serum 25(OH) D level was significantly inversely correlated with melanoma in Italy population [89]. Nevertheless, the evidence of the association of vitamin D with breast cancer risk remains controversial. Lowa [43] shown that low plasma vitamin D levels were related to higher breast cancer risk in the Caucasian population in the United Kingdom. However, McCullough [49] demonstrated that serum vitamin D level was not associated with the occurrence of breast cancer. Therefore, our meta-analysis aimed to explore the correct relationship between vitamin D and breast cancer risk.

In the current study, we performed a meta-analysis of 70 observational studies. No significant association of a 400 IU/day increment in vitamin D intake and breast cancer risk was observed. However, vitamin D intake might decrease the risk of breast cancer in Asian and premenopause women. In addition, the result of case-control studies indicated that there was an underlying linear relationship between blood vitamin D levels and the risk of breast cancer; the overall risk decreased by 6% for each 5 nmol/l increase in blood vitamin D.

The human body obtains a relatively small quantity of vitamin D through limited dietary sources; the major source, however, is endogenous production of vitamin D. Although the exact mechanism by which vitamin D is linked to breast cancer risk remains unclear, experimental studies reported an anti-proliferative effect of 1,25(OH)2D3 on malignant melanoma cells and a pro-differentiating effects on myeloid leukemia cells [90]. In addition, the recent experimental study indicated that 1,25(OH)_2_D3 played a long-lasting anti-inflammatory and anti-proliferation effect in synoviocytes of rheumatoid arthritis and osteoarthritis [91]. Furthermore, the ability of 1, 25(OH)2D3 to induce apoptosis and inhibit angiogenesis in cancer cells has been confirmed [9]. The experimental study has also demonstrated that 1, 25(OH)2D3 deficiency promotes tumorigenesis by increasing oxidative stress and DNA damage of malignant cells, and activating oncogenes and inactivating tumor suppressor genes, therefore enhancing cancer cells proliferation [92].

An anterior meta-analysis [13] showed that vitamin D intake exceeding 400IU/day was associated with a 8% reduction in breast cancer risk. However, a previous meta-analysis [93] including 10 prospective studies showed no association between vitamin D intake and breast cancer risk. The latest multicenter randomized double-blind placebo-controlled study also does not support the use of vitamin D supplementation in premenopausal women for breast cancer risk reduction [94]. In our meta-analysis of additional 9 studies [16, 17, 28–34], we received the same conclusion that the pooled OR of vitamin D intake 400 IU/day increment for breast cancer was 0.97 (95% CI = 0.92–1.02). However, when we stratified by geographical location, it tended to show a middle inverse association (OR = 0.89, 95%CI = 0.82–0.97) among Asian women and a strong opposite association (OR = 0.79, 95% CI = 0.64–0.96) for premenopausal women. Hence, our result did not support that vitamin D intake prevents breast cancer, except for Asian and premenopausal populations. Kawase [31] and Lee [32] both indicated that vitamin D intake decreased breast cancer risk in premenopausal women, and they also found that the anti-cancer mechanism of vitamin D centered on reproductive hormone and the higher level of serum reproductive hormone in premenopausal women may explain the relationship between vitamin D intake and premenopausal breast cancer risk. There may be specific vitamin D receptor gene polymorphisms associated with breast cancer risk in Asian women [95–97].

The recent meta-analysis [93] based on 24 observational studies about dietary and blood 25-hydroxyvitamin D (25(OH)D) included 31867 breast cancer cases showed that the RRs were 0.95 (95% CI = 0.88–1.01) for vitamin D intake and 0.92 (95% CI = 0.83–1.02) for blood 25(OH)D levels. An initial case-control study [10] showed a negative (OR = 1.00, 95% CI = 0.20–3.40) association between breast cancer risk and serum 1,25(OH)2 D levels > 51 pg/ml. However, a study [98] conducted in the Nurses' Health Study II, which researched circulating free 25(OH)D and risk of breast cancer, concluded the estimated risk of breast cancer associated with the high 25(OH)D level (OR = 3.2, 95% CI = 1.7–6.0). A recent large study conducted by Vojdeman [99], in 217244 individuals from Primary Health Care in Denmark, indicated that there was no association between an increment of 10nmol/l blood vitamin D levels and the incidence of breast cancer. In this meta-analysis, we have added more full studies, then came to an intuitive conclusion (OR = 0.94, 95% CI = 0.93–0.96) for blood vitamin D 5 units increases and low risk of breast cancer, and the dose-response curve shown a linear change (P_nonlinearity_ = 0**.**1893).

Significant heterogeneity was observed in this meta-analysis. Sensitivity analyses suggested that the stable pooled OR was not significantly affected by any single study. In addition, we performed subgroup analysis stratified by confounding factors to identify the sources of heterogeneity. Differences in baseline characteristics of the study population and vitamin D cut-off values may have also resulted in the observed heterogeneity.

The study had several limitations. Firstly, as most of the studies were designed as case-control, recall bias and selection bias were inevitable and affected the results. Secondly, our results showed a greater heterogeneity. We found distinct sources through subgroup analysis. The heterogeneity could be caused by the presence of different races, outdoor physical activities, season of blood collection, and the method for measuring blood vitamin D levels. Thirdly, breast cancer is a heterogeneous disease, and vitamin D may affect only some sub-types of breast cancer. However, there are only few studies on this topic and hence could not be included in the current meta-analysis. Lastly, there was an obvious publication bias, possibly because positive results are more likely to be published than negative results.

Compared with former studies, our meta-analysis included a greater number of single studies to increase the sample size. Hence, the results of this meta-analysis could be considered closer to reality. In addition, we used the exact dose-response to access the relation of blood vitamin D levels or vitamin D intake with breast cancer risk. This method gives us a more intuitive analysis of relationship between vitamin D and breast cancer.

In conclusion, our study supports the hypothesis that a higher blood vitamin D status is related to a lower risk of breast cancer. However, we cannot draw the same conclusion regarding the association between vitamin D intake and breast cancer risk. Further well-designed studies are needed to prove the results and to clarify the role of vitamin D against the pathogenesis of breast cancer.

## MATERIALS AND METHODS

### Literature retrieval

Relevant English publications up to January 2019 were searched in PubMed, EMBASE, and Web of Science using the search terms “Vitamin D,”, “25-hydroxyvitamin D,”, “25(OH)D,”, “breast neoplasms,”, “breast cancer,”, “incidence,” and “risk”. We also searched for relevant studies in the reference lists of the eligible meta-analysis and reviews. Two authors independently read the retrieved literature, screened the relevant publications according to the exclusion criteria, and then removed any duplication literature. Disagreement between two authors was resolved by discussion.

### Inclusion and exclusion criteria

Publications were screened according to the following inclusion criteria: (1) an original article; (2) a prospective cohort or case-control study in design; (3) the exposure factor was blood vitamin D levels or vitamin D intake; (4) the outcome of interest was the incidence of breast cancer; (5) availability of statistical parameters including the relative risk ratio (RR), hazard ratio (HR) or odds ratio (OR) with the corresponding 95% confidence intervals (CIs); alternatively, availability of sufficient data to calculate the aforementioned parameters; (6) ≥ three categories of exposure were provided. The exclusion criteria were the following: (1) a duplicate publication; (2) a publication which is not an original research such as reviews and systematic reviews; (3) low quality of research and poor reliability.

We chose total vitamin D intake when the study reported results on both dietary and total vitamin D intake. The RR, HR, with the corresponding 95% CI were recalculated when the low category of vitamin D was not the reference category in the original study.

### Data extraction and quality assessment

From each selected publication, the following variables were collected: name of the first author, year of publication, geographical location where the study was conducted, type of study (case-control or cohort), follow-up periods, age of the study population, number of cases and controls, person-time, source of measurement of vitamin D levels (serum or plasma), source of vitamin D intake (dietary or dietary and supplement), blood vitamin D levels (nmol/l), vitamin D intake (IU/day), HR, RR or OR, 95% CI, and confounding factors that were adjusted. If a study provided several risk estimates, we used the estimate from the major multivariable model, which included a greater number of adjusted confounders. To facilitate comparison, the value of blood vitamin D levels and vitamin D intake that were expressed in conventional units (ng/ml and ug/d, respectively) were converted to SI units (nmol/l and IU/day, respectively).

The quality of each selected publication was assessed independently by two authors according to the Newcastle-Ottawa Quality Assessment Scale (NOS) [100]. The content of the study was evaluated for four major aspects: selection, comparability, exposure, and results, and thereafter, categorized into high, medium, and low quality. A study with a score > 6 was considered to be well quality.

### Statistical analysis

We used the Q or I^2^ statistics to assess the heterogeneity among the studies [101]. P < 0.1 or I^2^ > 50% indicated significant heterogeneity. The random-effects model was used to estimate the pooled ORs and 95% CIs for a 5 -unit increment in blood vitamin D levels and a 100 -unit increment in vitamin D intake [102]. We evaluated the mean of the natural logarithm of the ORs, and weighted the OR of each study by the reciprocal of its variance. All statistical tests were two-sided, P < 0.05 was considered significance.

In all studies on the three categories of data on vitamin D, we assigned a median value of vitamin D to each category. For the open-ended upper category, we assumed that it had the same amplitude as the previous one. The generalized least square for tend estimation was used to transfer category-specific risk values to the OR related to every 5nmol/l and 400IU/day increase in blood vitamin D levels and vitamin D intake, respectively [103].

We used a two-stage hierarchical regression model to examine the possible linear dose-response association between blood vitamin D levels or vitamin D intake and breast cancer risk [104]. We analyzed data using the random-effects restricted cubic spline and four knots models. The covariance of multivariate adjusted OR was estimated by using the methods of Greenland and Longnecker [105].

We conducted subgroup analyses based on menopausal status, study type, geographical location, vitamin D source and follow-up duration. Sensitivity analyses were performed to assess the effect of individual study on the results. Potential publication bias was assessed by using Egger’s test and Begg’s test. All data were analyzed using the Stata 12 software.

## Supplementary Material

Supplementary Figures

Supplementary Table 1
