# Supplementary material for: Vitamin D intake, blood vitamin D levels, and the risk of breast cancer: a dose-response meta-analysis of observational studies
Source: Aging (Albany NY). 2019 Dec 28;11(24):12708–32. doi: 10.18632/aging.102597 (PMC6949087; doi:10.18632/aging.102597)
Supplement: Supplementary Figures [file aging-11-102597-s002..pdf]

SUPPLEMENTARY FIGURES

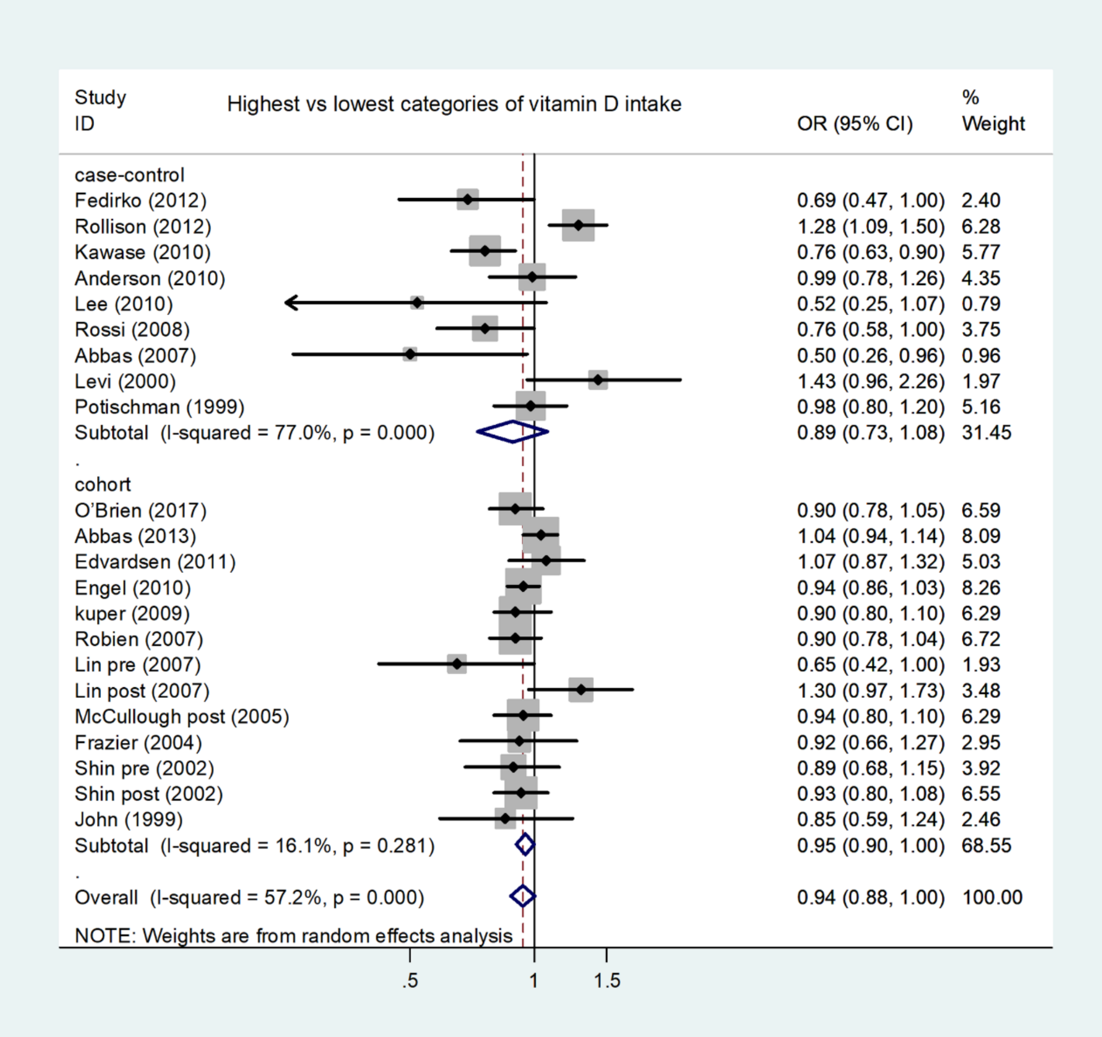

Supplementary Figure 1. Forest plot of meta-analysis of breast cancer risk in relation to Highest vs lowest categories of vitamin D intake. Abbreviations: OR, odds ratio; CI, confidence interval.

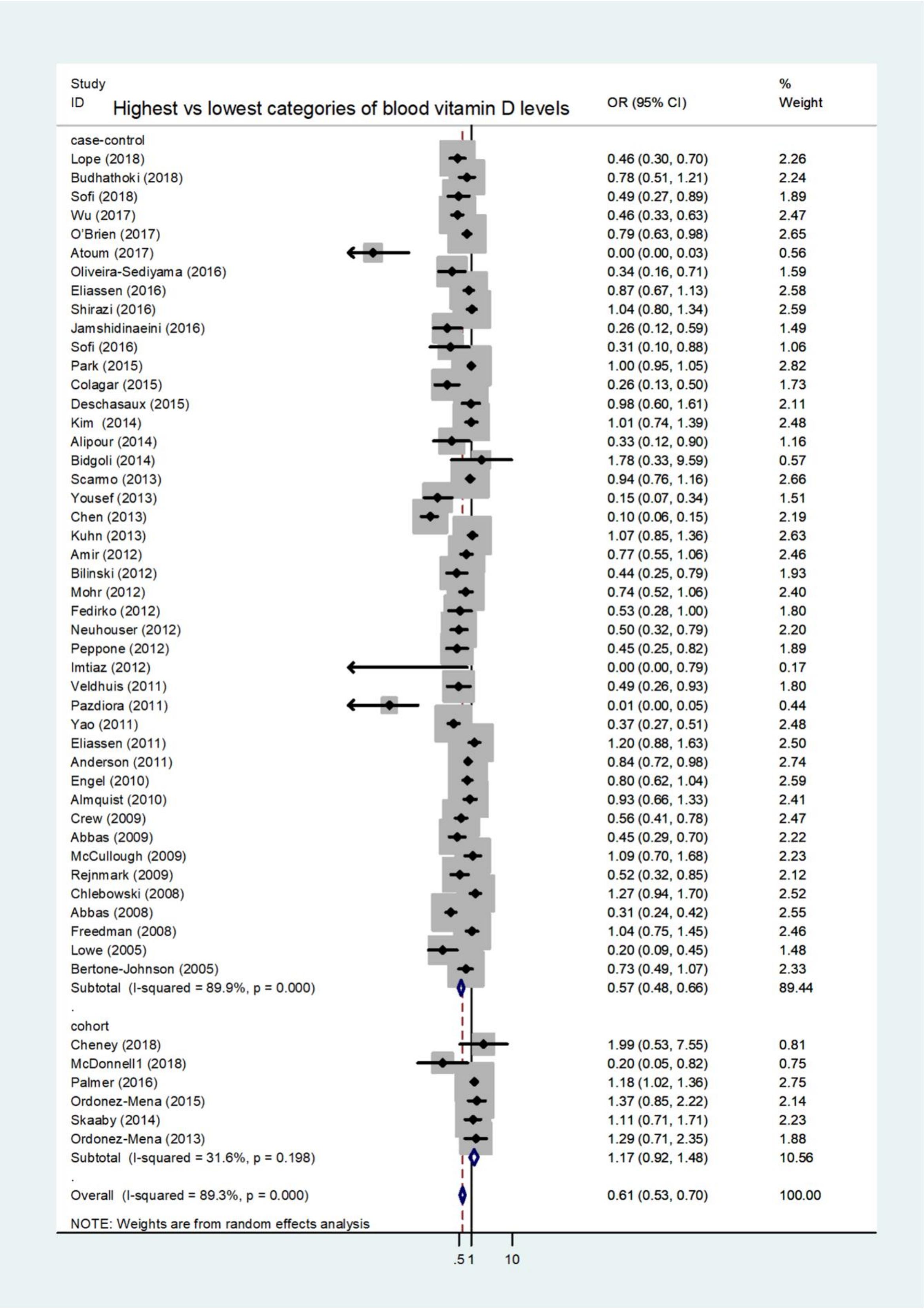

Supplementary Figure 2. Forest plot of meta-analysis of breast cancer risk in relation to Highest vs lowest categories of blood vitamin D. Abbreviations: OR, odds ratio; CI, confidence interval.

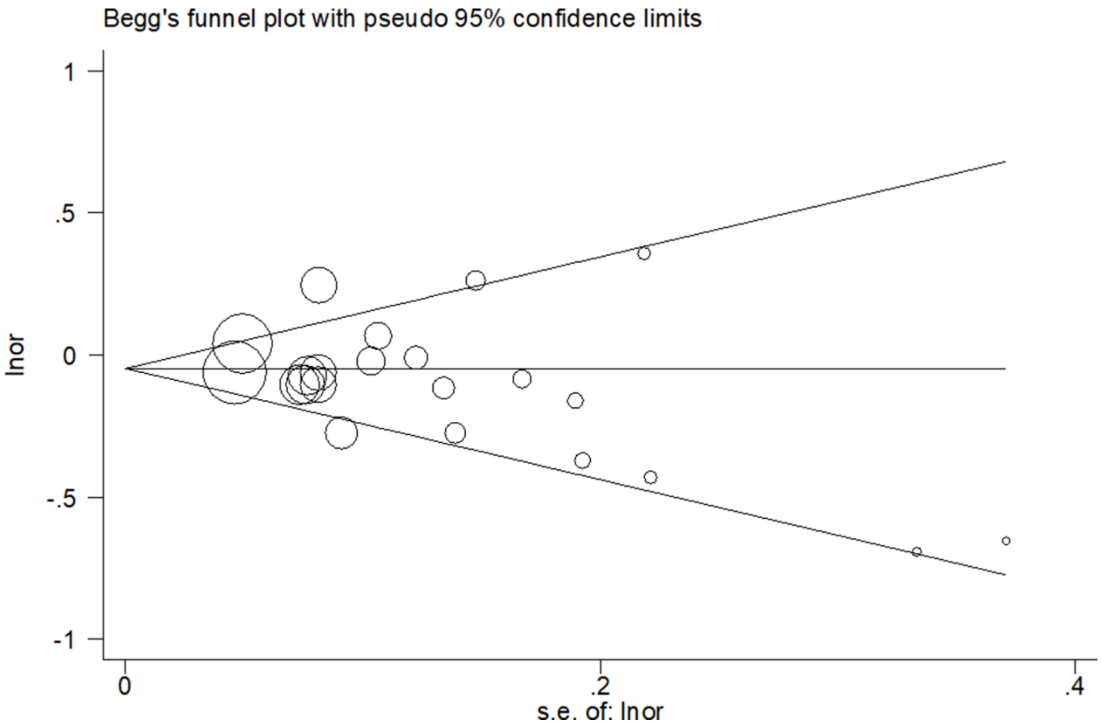

Supplementary Figure 3. Funnel plot of meta-analysis of breast cancer risk in relation to Highest vs lowest categories of vitamin D intake.

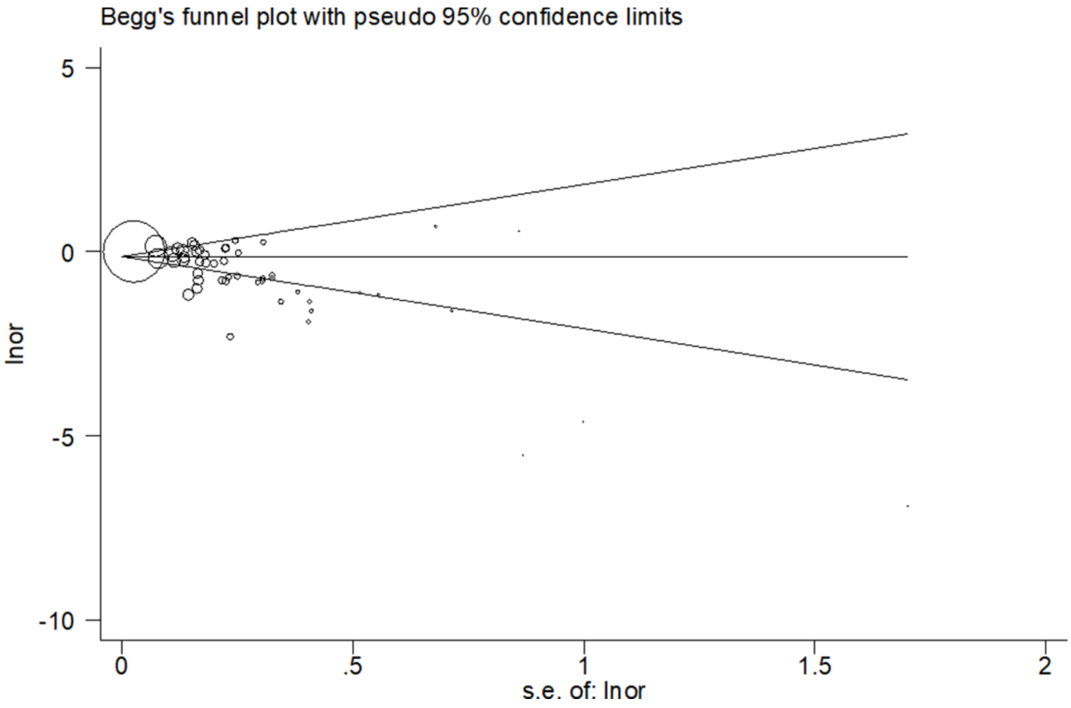

Supplementary Figure 4. Funnel plot of meta-analysis of breast cancer risk in relation to Highest vs lowest categories of blood vitamin D.

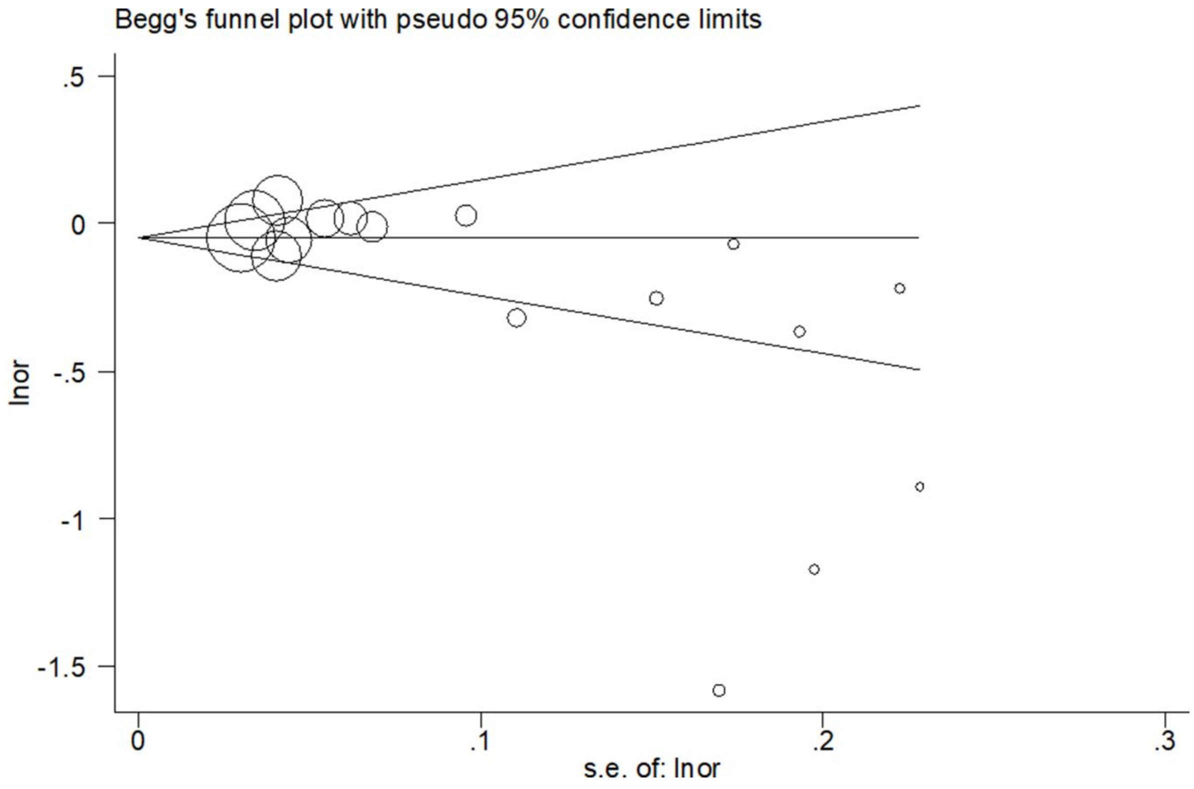

Supplementary Figure 5. Funnel plot of meta-analysis of breast cancer risk in relation to vitamin D intake increment (per 400IU/day).

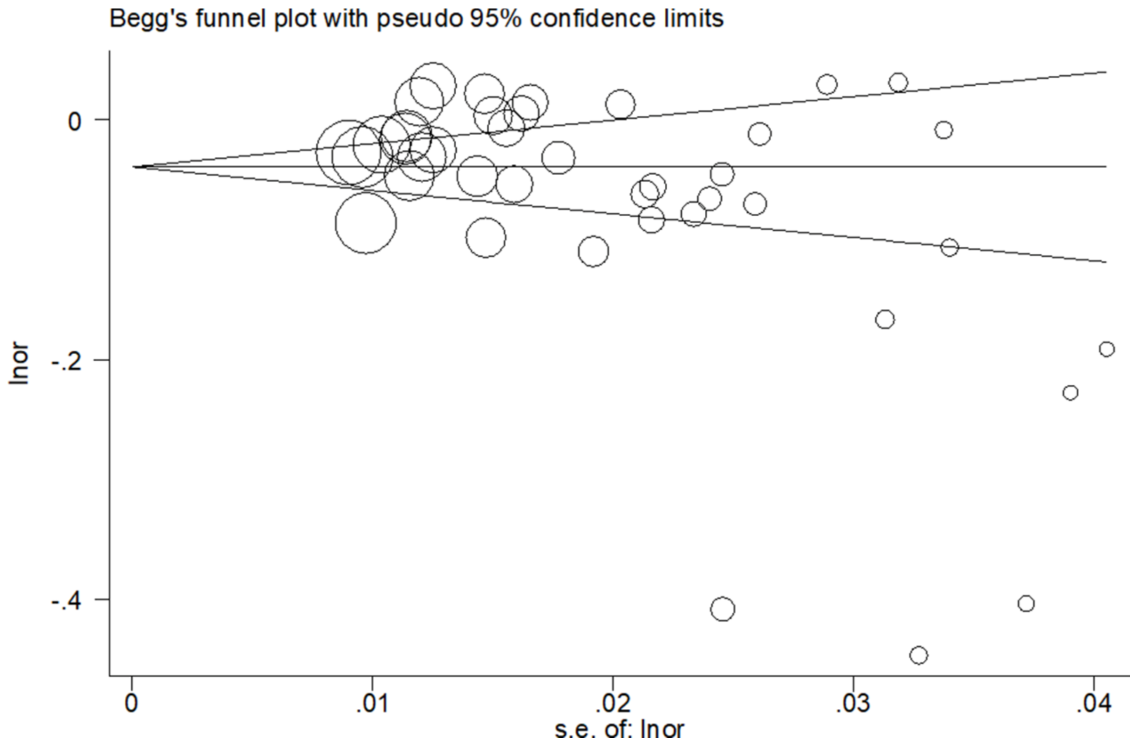

Supplementary Figure 6. Funnel plot of meta-analysis of breast cancer risk in relation to blood vitamin D increment (per 5nmol/l).

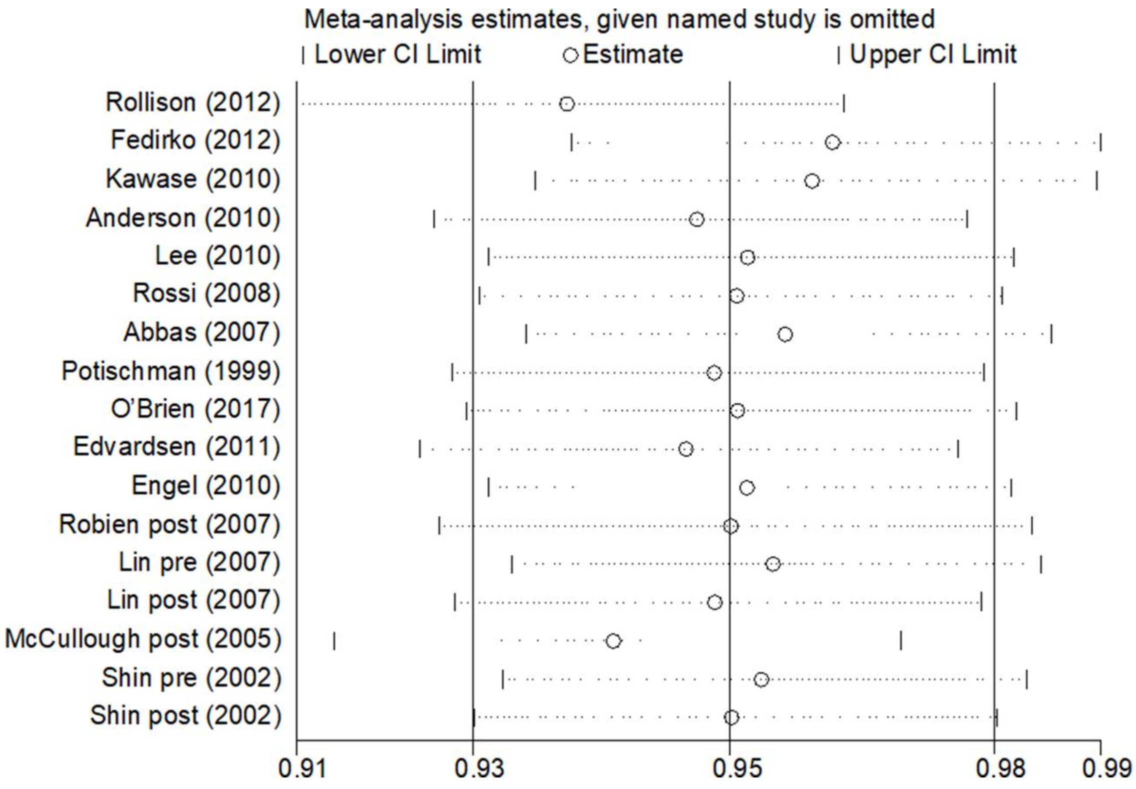

Supplementary Figure 7. Sensitivity analysis of meta-analysis of breast cancer risk in relation to vitamin D intake increment (per 400IU/day).

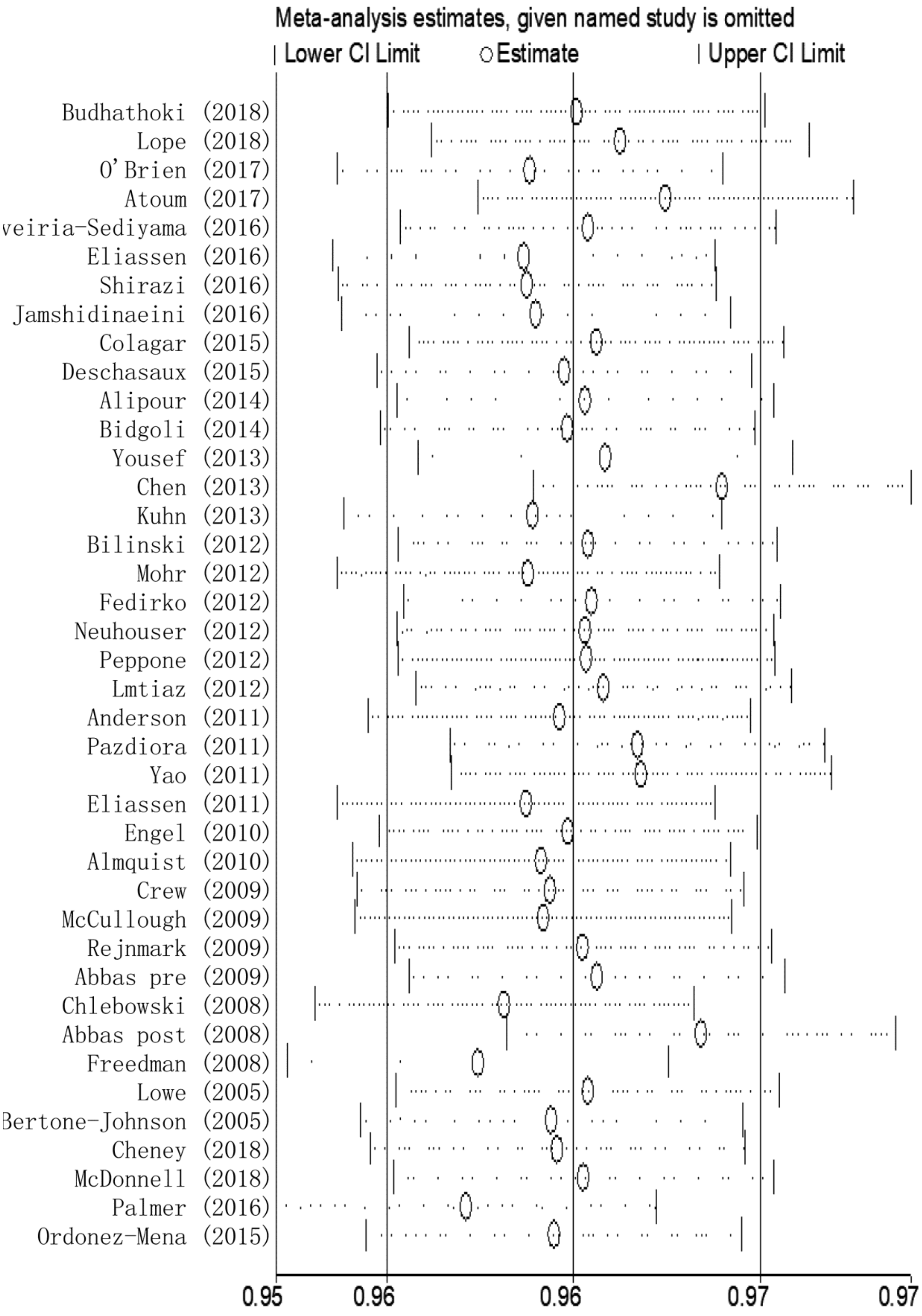

Supplementary Figure 8. Sensitivity analysis of meta-analysis of breast cancer risk in relation to blood vitamin D increment (per 5nmol/l).
