# Supplementary material for: Vitamin D intake, blood vitamin D levels, and the risk of breast cancer: a dose-response meta-analysis of observational studies
Source: Aging (Albany NY). 2019 Dec 28;11(24):12708–32. doi: 10.18632/aging.102597 (PMC6949087; doi:10.18632/aging.102597)
Supplement: Supplementary Table 1 [file aging-11-102597-s001..docx]

| **Supplementary Table 1. Characteristics of prospective studies included in the meta-analysis of blood vitamin D and breast cancer risk.** | | | | | | | | |
| --- | --- | --- | --- | --- | --- | --- | --- | --- |
| **Author** | **Country** | **Study type** | **Follow-up period (year)** | **Age (year)** | **No. of cases/controls** | **Measure of exposure level**  **(nmol/l)^a^** | **Adjusted OR(95%CI)** | **Adjustment factors** |
| Cheney et al, 2018 | German | Cohort | 7 years | cancers 59.7  non-cancers 53.3 | 16/993 | Serum:  45-183.25 vs 5-28.25 | 1.99  (0.53–7.55) | Age, BMI, season of blood draw, menopausal status, physical activity, alcohol consumption, smoking status, and vitamin D supplementation |
| Lope et al, 2018 | Spain | Case- control | 2008-2013 | 20-85 | 546/558 | Serum: >63.70 vs ≤29.98 | 0.46  (0.30-0.70) | Age, BMI, geographical area, menopausal status and day of sample extraction, educational level, ethnicity, age at first full term delivery, family history of breast cancer, previous breast biopsies, HRT use, skin color, hypercholesterolemia, and physical activity in the last 5 . |
| Sofi et al, 2018 | India | Case- control | 2015-2017 | 25-85 | 200/200 | Serum: ≤50 vs >50 | 2.40  (1.20-5.10)^b^ | Unknown |
| McDonnell et al, 2018 | USA | Cohort | 4 years | 59-69 | 77/5038 | Serum: ≥150 vs <50 | 0.20  (0.05-0.82) | Age, BMI, smoking status, and calcium supplement intake |
| Budhathoki et al, 2018 | Japan | Case- control | 1993-2009 15.9 years | 40-69 | 212/4044 | Plasm: 72.6 vs 36.9 | 0.78  (0.51-1.21) | Age, BMI, smoking, alcohol use, physical activity, family history of cancer, and reported history of diabetes, age at menarche, number of births, use of exogenous female hormones, and menopausal status and age at menopause |
| O’Brien et al, 2017 | USA | Case- control | 2003-2009 5years | 35-74 | 1600/1822 | Serum: >36.8 vs ≤24.6 | 0.79  (0.63-0.98) | Age, BMI, race, education level, current hormonal birth control use, current hormone therapy use and type, physical activity, alcohol consumption, osteoporosis, parity, and a BMI× menopausal status interaction term. |
| Atoum et al,2017 | Jordanian | Case-  control | 2013-2015 | 40-69 | 122/100 | serum: >62.5 vs <25 | 0.03  (0.004- 0.22) | Rude |
| Wu et al, 2017 | USA  Hispanic | Case-  control | 1995-2007 | 20-80 | 243/417 | Serum: <50 vs ≥50 | 2.0  (1.40-3.0)^b^ | Age, BMI, ethnicity, and the seasons of blood draw. |
| Sofi et al, 2016 | India | Case-  control | 201-2015 | Cases 45±9  Controls 46±10 | 100/100 | Serum: ≤50 vs >50 | 2.50  (0.90-7.40)^b^ | Unknown |
| Palmer  et al, 2016 | USA | Cohort | 1995-2013 | 21-69 | 1454/59000 | Serum: ＜21vs﹥40 | 1.23  (1.04-1.46)^b^ | Age, BMI, family history of breast cancer, age at menarche, age at menopause, age at first birth, parity, oral contraceptive use, and use of estrogen and progesterone female hormones |
| Oliveira-Sediyama  et al, 2016 | Brazil | Case- control | unknown | 44-59 | 197/181 | Serum: ≥75 vs <50 | 0.34  (0.16-0.71) | Rude |
| Eliassen et al, 2016 | USA | Case- control | 1989-2010 | 43-69 | 1506/1506 | Plasm: ≥81.75 vs <43.75 | 0.87  (0.67–1.13) | Age at menarche, BMI at age 18, weight change from age 18 to blood draw, parity and age at first birth, family history of breast cancer, history of benign breast disease |
| Shirazi et al,2016 | Sweden | Case- control | 1991-2005 | 57 | 764/764 | Serum: ≥98 vs ≤76 | 1.04  (0.80-1.34) | Age, and menopausal status |
| Jamshidinaeini  et al, 2016 | Iran | Case- control | 2013-2014 | 50 | 135/135 | Serum: 73.75-349.75 vs 2.25-25.75 | 0.26  (0.12-0.59) | Age, BMI, use of exogenous hormones, education, menopause status, duration of sun exposure, calories, fat, and calcium intake |
| Deschasaux  et al, 2015 | France | Case- control | 1994–2007 | Cases 49.3  Controls 49.1 | 233/466 | Plasma: ≥58.75 vs <28.5 | 0.98  (0.60-1.61) | BMI, educational level, physical activity, alcohol intake, smoking status, height, family history of breast cancer, and number of biological children. |
| Ordonez-Mena  et al, 2015 | Europe | Cohort | 1992–2000 | ≥60 | 378/7813 | Serum: <30 vs >50 | 0.73  (0.45-1.18)^b^ | Age, BMI, sex, season of blood draw, education, smoking status, and vigorous physical activity |
| Colagar et al, 2015 | Iran | Case- control | 2009–2013 | Cases 48.7  Controls 47 | 117/113 | Serum: ≤22.5 vs >40 | 3.90  (1.99-7.61)^b^ | Rude |
| Park et al, 2015 | Korea | Case- control | 2006-2012 | Cases 50.7±10.5  Controls49.7±16.3 | 3634/17133 | Serum: <50 vs ≥50 | 1.28  (1.15-1.43)^b^ | Age, BMI, residential regions, marriage status, age at menarche, menopausal status, age at first birth, and HRT use |
| Alipour et al, 2014 | Iran | Case- control | unknown | Cases 44.2  Controls 43.2 | 136/364 | Serum: <31.25 vs >87.75 | 3.00  (1.11-8.1)^b^ | Age, age at first birth, menarche, menopausal status, parity, history of breastfeeding, and family history of breast cancer |
| Kim et al, 2014 | USA | Case- control | 2001-2006 | Cases 68.5  Controls 68.4 | 680/580 | Serum: <50 vs ≥50 | White 7.5(1.41-39.8)^b^  Africa-American 0.74(0.36-1.53)^b^  Hawaiian 0.74(0.13-4.29)^b^  Japanese 0.96(0.47-1.97)^b^  Latino 0.9(0.41-1.95)^b^ | Age, BMI, season, sunburn history, strenuous sports, number of live births, family history of breast cancer, use of multivitamin, and use of calcium supplement. |
| Bidgoli et al, 2014 | Iran | Case-  control | 2010-2012 | Cases 36.45±7.02 Controls 34.2±5.7 | 60/116 | serum: 75-125 vs <12.5 | 1.78  (0.33 - 9.59) | Rude |
| Skaaby et al, 2014 | Denmark | Cohort | 1993-2008  (11.3) | 18-71 | 159/5606 | Serum Q4 vs Q1 | 1.11  (0.71-1.71) | BMI, study, gender, education, season, physical activity, smoking habits, alcohol intake, and intake of fish. |
| Ordonez-Mena  et al, 2013 | Germany | Cohort | 2000-2002 | 50-74 | 137/5261 | Serum Q4 vs Q2+Q3 | 1.39  (0.89–2.18) | Age, BMI, sex, multivitamin use, fish consumption, red meat consumption, daily fruit intake, daily vegetables intake, scholarly education, physical activity, smoking, and family history of cancer. |
| Yousef et al, 2013 | Saudi Arabia | Case- control | unknown | 18-75 | 120/120 | Serum: <25 vs ≥50 | 6.10  (2.40-15.1)^b^ | Age, BMI, parity, education, history of breastfeeding, and walking outdoors. |
| Chen et al, 2013 | China | Case- control | 2006-2007 | 30-87 | 593/580 | Serum: >44.75 vs ≤26.0 | 0.10  (0.06-0.15) | Age, age at first birth, age at menarche, use of contraceptive, menopausal status, first-degree relatives’ history of breast cancer and season of blood collection. |
| Kuhn et al, 2013 | Europe | Case- control | 1992-2006 | 35-70 | 1391/1391 | Plasm: >63.0 vs ≤39.3 | 1.07  (0.85-1.36) | BMI, age at first period, age at first full-term pregnancy, number of full-term pregnancies, breastfeeding, alcohol consumption, smoking status, education level and physical activity. |
| Mohr et al, 2013 | USA | Case- control | 1994-2009 | 39.6 | 600/600 | Serum: ≤37.25 vs ≥88.0 | 1.19  (0.80-1.8)^b^ | Rude |
| Scarmo et al, 2013 | USA  Sweden | Case- control | 1985-2007  1995-2010 | 34-69 | 1585/2940 | Serum Q5 vs Q1 | 0.94  (0.76-1.16) | BMI, age at menarche, age at first birth/parity, family history of breast cancer, HRT use, and alcohol consumption. |
| Amir et al, 2012 | Canada | Case- control | 1992-1997 | 53.6 | 231/856 | Serum 86 vs 31 | 0.81  (0.58–1.13) | Rude |
| Bilinski et al, 2012 | Australia | Case- control | 2008-2010 | 55.4 | 214/852 | Serum: <25 vs ≥75 | 2.30  (1.30 - 4.3)^b^ | Rude |
| Fedirko et al, 2012 | Mexico | Case- control | 2004-2007 | 35-69 | 573/639 | Serum: >75 vs <50 | 0.53  (0.28-1.00) | Age, BMI, region, health care system, season of blood draw, alcohol consumption, total energy intake, family history of breast cancer, parity/number of children born alive, age at first full term pregnancy, breast-feeding, use of hormone for menopause, height, physical activity index, and menopausal status |
| Neuhouser et al, 2012 | USA | Case- control | 1994-2005 | 50-79 | 1080/1080 | Serum: <36.7 vs ≥64.9 | 1.06  (0.78-1.43)^b^ | BMI, smoking, WHI intervention arm, physical activity, mammography within the past 2 , Gail 5-year risk score, HRT use, and alcohol intake |
| Peppone et al, 2012 | USA | Case- control | 2009-2010 | 40-70 | 194/194 | Serum: <50 vs ≥80 | 2.41  (1.30-4.48)^b^ | Age, laboratory, and month of blood draw |
| Lmtiaz et al, 2012 | Pakistan | Case-  control | 2010-2011 | Cases 47.5±9.8 Controls 46.2±2.6 | 90/90 | Serum: >100 vs <50 | 0.001  (0.001-0.79） | Rude |
| Pazdiora et al, 2011 | Czech Republic | Case- control | unknown | 63 | 43/214 | Serum: >62.5 vs <37.5 | 0.01  (0.001-0.05) | Rude |
| Veldhius et al, 2011 | Netherland | Case- control | 2004-2009 | Cases 67.6  Controls 62.4 | 56/829 | Serum: <50 vs ≥50 | 1.43  (0.84-2.44)^b^ | Rude |
| Yao et al, 2011 | USA | Case- control | 2003-2008 | unknown | 579/574 | Serum: ≥75 vs <50 | 0.37  (0.27-0.51) | Age and BMI |
| Anderson et al, 2011 | Canada | Case-  control | 2003-2004 | 56 | 3101/3471 | serum:  73.3–87 vs 39–59.9 | 0.84  (0.72 -0.98) | Age |
| Eliassen et al, 2011 | USA | Case- control | 1996-2007 | 32-54 | 613/1218 | Serum: ≥76.4 vs <45.9 | 1.20  (0.88-1.63) | Age at menarche, BMI at age 18, parity and age at first birth, BMI at blood collection, family history of breast cancer, and history of benign breast disease. |
| Engel et al, 2010 | France | Case- control | 10 | 56.9 | 636/1272 | Serum: >67.5 vs <49.5 | 0.80  (0.62-1.04) | Age, menopausal status at blood collection, age at menopause, age at menarche, study center, date of blood collection, BMI at the time of blood collection, physical activity, number of children, tobacco status, previous use of oral contraceptives, MHT use, personal history of mammography, benign breast disease, and previous family history of breast cancer |
| Almquist et al, 2010 | Sweden | Case- control | 1991–2006 | 57 | 764/764 | Serum: ≥106 vs <70 | 0.93  (0.66-1.33) | BMI, educational level, socioeconomic index, alcohol consumption, smoking status, marital status, country of birth, age at menarche, use of oral contraception, number of children, HRT-use, continuous values of albumin, quartiles of 25OHD, PTH and calcium, and creatinine and phosphate. |
| Crew et al, 2009 | USA | Case- control | unknown | Cases 58.6 Controls 56.1 | 1026/1075 | plasm: >100 vs <50 | 0.56  (0.41-0.78) | Age, BMI, race, age of menarche, age of first birth, parity, HRT use, breastfeeding history, menopausal status, first-degree family history of breast cancer, history of benign breast disease, physical activity, and season of blood draw. |
| McCullough et al, 2009 | USA | Case- control | 1999–2005(6.9) | 47–85 | 516/516 | Serum: >73.2 vs <36.7 | 1.09  (0.70-1.68) | Birth year, year of blood draw, season, race, parity and age at first birth, BMI at blood collection and weight change from age 18 to blood collection |
| Rejnmark et al, 2009 | Denmark | Case- control | 2003–2007 | 58 | 142/420 | Plasm: >84 vs <60 | 0.52  (0.32-0.85) | Rude |
| Abbas et al, 2009 | Germany | Case- control | unknown | 30–50 | 289/595 | Plasma: ≥60 vs <30 | 0.45  (0.29-0.70) | Age, BMI, number of births, first-degree family history, age at menarche, duration of breast-feeding, and alcohol consumption |
| Abbas et al, 2008 | Germany | Case- control | 2001-2005 | 50–74 | 1394/1365 | Serum: ≥75 vs <30 | 0.31  (0.24-0.42) | BMI, time of blood collection, year of birth, adjusted for age at menopause, first-degree family history of breast cancer, history of benign breast disease, number of pregnancies, age at menarche, breastfeeding history, total number of mammograms, use of HT, education level, smoking status |
| Chlebowski et al, 2008 | USA | Case- control | 7 | 50–79 | 895/898 | Serum: <32.4 vs ≥ 67.6 | 1.22  (0.89-1.67)^b^ | Age, BMI, race, latitude of clinical center, venipuncture date, hormone therapy, family history of breast cancer, history of breast biopsy, current estrogen plus progestin use, and current estrogen-only use |
| Freedman et al, 2008 | USA | Case- control | 4–8.5 | 55-74 | 1005/1005 | Serum: ≥84.25 vs <45.75 | 1.04  (0.75-1.45) | Age, BMI, period of blood draw, age at menarche, season of serum collection, age at menopause, HRT use, benign breast disease, family history of breast cancer, combination of parity and age at first birth, smoking status, daily alcohol intake, and daily dietary calcium intake |
| Lowe et al, 2005 | UK | Case- control | 1998-2003 | 34-84 | 179/179 | Plasm: <50 vs ≥150 | 5.83  (2.31-14.7)^b^ | Rude |
| Bertone-Johnson  et al, 2005 | USA | Case- control | 1989-1996 | 43–69 | 701/724 | Plasma: ≥100 vs ≤50 | 0.73  (0.49-1.07) | Matching factors, BMI at age 18, parity/age at first birth, family history of breast cancer, history of benign breast disease, postmenopausal hormone use, age at menarche, age at menopause, alcohol intake, and plasma a-carotene. |

Abbreviations: OR=odds ratio; CI=confidence interval; BMI=body mass index (kg/m^2); HRT=hormone replacement therapy; HT=hormone therapy; MHT= menopausal hormone therapy; WHI=Women’s Health Initiative.

1. Blood vitamin D levels in ng/ml were converted to nmol/l using the conversion factor, 1 ng/ml=2.5 nmol /l.
2. The ORs of eleven studies that used the highest category of blood vitamin D levels as a reference were recalculated using the lowest category as a reference to be included in the meta-analysis.
